# *Bacillus subtilis* Fermentation of *Malva verticillata* Leaves Enhances Antioxidant Activity and Osteoblast Differentiation

**DOI:** 10.3390/foods9050671

**Published:** 2020-05-22

**Authors:** Keumok Moon, Seola Lee, Jaeho Cha

**Affiliations:** 1 Department of Microbiology, Pusan National University, Busan 46241, Korea; moonko81@nate.com (K.M.); lsa2715@pusan.ac.kr (S.L.); 2 Microbiological Resource Research Institute, Pusan National University, Busan 46241, Korea

**Keywords:** *Malva verticillata*, *Bacillus subtilis*, fermentation, osteoblast differentiation

## Abstract

*Malva verticillata*, also known as Chinese mallow, is an herbaceous plant with colorful flowers and has been used as a medicine for thousands of years. This study investigated this herb for potential antioxidant activity or an association with osteoblast differentiation. *M. verticillate* leaves were fermented with *B. subtilis* MV1 at 30 °C for 7 days to enhance their biological activities. The resultant aqueous extract (MVW) and the fermented leaves (MVB) were measured for antioxidant and osteoblast differentiation. The results showed that the total phenolic, flavonoid, and antioxidant activity, as well as the osteoblast differentiation of the MVB increased (2 to 6 times) compared with those of the MVW. MVB induced phosphorylation of p38, extracellular signal-regulated kinase in C3H10T1/2 cells, and the phosphorylation was attenuated via transforming growth factor-β (TGF-β) inhibitors. Moreover, runt-related transcription factor 2 and osterix in the nucleus increased in a time-dependent manner. The messenger RNA expression of alkaline phosphatase and bone sialoprotein increased about 9.4- and 65-fold, respectively, compared to the non-treated cells. MVB stimulated C3H10T1/2 cells in the osteoblasts via TGF-β signaling. Thus, fermented *M. verticillata* extract exhibited enhanced antioxidant activity and osteoblast differentiation.

## 1. Introduction

Osteoporosis is a skeletal disorder characterized by reduced bone mineral density and mass, resulting in damaged bone structure. This condition is a silent disease until fractures occur, leading to serious secondary health problems, and even death. Factors that affect skeletal fragility include aging, genetics, nutrition, vitamin and mineral deficiency, lifestyle choices, smoking history, hormone production, and medications [1]. Osteoporosis therapeutic agents include bone resorption inhibitors (which are the primary therapy) and also bone formation stimulators in the osteoblasts. The osteoblasts can differentiate from the mesenchymal stem cells (MSC), via several processes involving the bone morphogenetic protein (BMP) and parathyroid hormone [2]. Mature osteoblasts release bone matrix protein, including collagen type I, osteocalcin, osteopontin, osteonectin, and alkaline phosphatase (ALP). These cells mineralize almost the entire bone matrix to create new bone; thus, loss of osteoclasts due to aging impairs healing of fractures.

*Malva verticillata* is an annual or biennial plant that grows in temperate and subtropical regions and belongs to the Malvaceae family [3]. Mature seeds of *M. verticillata* have been used as medicines for centuries as diuretics and laxatives [4]. Recently, a water extract of *M. verticillata* seeds was reported to inhibit osteoclastogenesis and bone resorption by suppressing the receptor activator of the NF-κB ligand (RANKL) signaling pathway without affecting osteoblast differentiation [5]. Ethanol extracts from the *M. verticillata* seeds increased the Wingless-related integration site (Wnt) activity in a concentration-dependent manner and led to increased β-catenin levels in cultured human dermal papilla cells (DPCs) [6]. A study by Shim et al. evaluated *Malva verticillata* with Gas Chromatography and Mass Spectrometry (GC-MS) analysis and found that this herb contains the following chemical compounds, including 1,3-dihydroxyacetone dimer, d-alanine, 5-hydroxymethyl furfural, 2-hydroxygamma-butyrolactone, palmitic acid, oleamide, and β-sitosterol [5]. Moreover, myristoleic acid, a compound found in the seeds, stimulates the proliferation of DPCs in a dose-dependent manner and increases the transcriptional levels of the downstream targets such as insulin-like growth factor 1, vascular endothelial growth factor, and hepatocyte growth factor [6]. Furthermore, the leaves, stems, and seeds of *M. verticillata* have been shown to be a rich source of phenolic compounds. Specifically, the leaves contained various flavonoids and their derivatives, which were ideal for 2,2′-azino-bis (3-ethylbenzothiazoline-6-sulfonic acid) (ABTS) free radical scavenging and possessed ferric reducing antioxidant power [7].

Microbial fermentation has been used to increase the extraction yield of bioactive compounds from natural products or to produce new compounds [8,9,10]. Bacteria such as *Bacillus subtilis* and *Lactobacillus pentosus*, and fungi such as *Aspergillus oryzae* or *Rhizopus oryzae*, as well as yeasts such as *Saccharomyces* spp., *Pichia* spp., and *Brettanomyces* spp. have been used for the conversion of inexpensive compounds into useful and valuable compounds [11,12,13,14,15,16]. *B. subtilis* has been used for the production of recombinant proteins and chemicals because it effectively grows on cheap carbon sources, possesses clear inherited backgrounds, has mature genetic manipulation methods, and exhibits robustness in large-scale fermentations [17]. Chen et al. used *B. subtilis* B7-S for the production of vanillin, one of the most important flavor compounds, derived from ferulic acid [11]. Dajanta et al. reported that black and yellow soybeans fermented with *B. subtilis* possess enhanced phenolic and flavonoid content as well as antioxidant activity [18].

This study measured osteoblast differentiation of the aqueous extracts of *M. verticillata* leaves. *B. subtilis* MV1 was isolated from *M. verticillata* leaves and used to enhance the osteoblast differentiation of the aqueous extracts. The osteoblast differentiation and antioxidant activities of the aqueous extracts fermented with *B. subtilis* MV1 were compared with non-fermented aqueous extracts.

## 2. Materials and Methods

### 2.1. Materials

*M. verticillata* leaves were purchased from a local market in Busan, Korea, in July 2018. The leaves were washed with tap water to remove dirt particles on the surface and then dried at 60 °C for 12 h. The leaves were ground using a commercial blender and then kept frozen until use. Glucose, gallic acid, quercetin dihydrate, Folin-Ciocalteu reagent, aluminum chloride, dimethyl sulfoxide (DMSO), 2,2-diphenyl-1-picrylhydrazyl (DPPH), 2,4,6-Tris(2-pyridyl)-1,3,5-triazine (TPTZ), 3-(4,5-dimethylthiazol-2-yl)-2,5-diphenyltetrazolium bromide (MTT), and β-glycerolphosphate were purchased from Sigma-Aldrich (St. Louis, MO, USA). Sodium carbonate, potassium acetate, ascorbic acid, and iron (III) chloride hexahydrate were purchased from Junsei Chemical Co., Ltd. (Tokyo, Japan). The methanol was of a low particulate grade and purchased from SK Chemicals (Ulsan, Korea). Yeast-peptone-dextrose (YPD) and malt extract broth (MEB) were purchased from Difco (Sparks, MD, USA). Minimum Essential Media-α (MEM-α) and Dulbecco’s Modified Eagle Medium (DMEM) were purchased from WelGENE Inc. (Daegu, Korea), and fetal bovine serum (FBS) and antibiotics (penicillin/streptomycin) were purchased from Gibco (Thermo Fisher Scientific, Waltham, MA, USA). Bone morphogenetic protein-2 (BMP-2) was provided from Cowellmedi Co., Ltd. (Busan, Korea). Then the following inhibitors, U0126, SB525334, and SB203580 (Promega corp., Madison, WI, USA), were added into the differentiation medium (at 10 µM), respectively to inhibit the molecular signaling activities.

### 2.2. Extraction of the M. verticillata Leaves

The extraction process of the *M. verticillata* leaves involved the following: 10 g of the frozen ground leaves was added into 200 mL of distilled water or methanol, followed by sonication for 60 min at 50 °C. The supernatant was collected, and this process was repeated three times. The supernatant that was obtained each time was then mixed and filtered (No. 2, Toyo Roshi Kaisha Ltd., Tokyo, Japan). The filtrate was concentrated with a rotary vacuum evaporator at 60 °C and lyophilized. Then the aqueous extracts (MVW, 3.9 g) and the methanol extracts (MVM, 2.4 g) were kept frozen until use.

### 2.3. Isolation and Identification of the Bacteria from M. verticillata Leaves

The dried *M. verticillata* leaves were used to isolate microorganisms. The powder of the leaves was spread onto an YPD and Luria–Bertani (LB) agar plate and cultured overnight at 30 °C. The isolates were cultivated in LB broth, and then, DNA was extracted from the cells. The 16S ribosomal RNA region was amplified by PCR using the universal primers 27F (5′-AGAGTTTGATCMTGGCTCAG-3′) and 1492R (5′-TACGGYTACCTTGTTACGACTT-3′) and sequenced with the primers 785F (5′-GGATTAGATACCCTGGTA-3′) and 907R (5′-CCGTCAATTCMTTTRAGTTT-3′, Macrogen Inc., Seoul, Korea). Resultant 16S rDNA gene sequences were compared using the BLAST program (version 2.6.0) to identify the genus and species of the isolated bacterium.

### 2.4. Preparation of M. verticillata Leaf Fermented Aqueous Extracts

#### 2.4.1. Biotransformation of *M. verticillata* Leaves by *Bacillus* spp.

*Bacillus subtilis* MV1, *B. velezensis* MV2, and *B. subtilis* 168 were grown at 30 °C on an LB agar plate. One gram of *M. verticillata* leaves was added to 200 mL of LB broth, sonicated for 60 min, and then, 2 mL of *Bacillus* (OD_600 nm_ = 1) was inoculated into the broth. Cultivation was carried out at 30 °C. Following cultivation for 7 days, the medium was separated by centrifugation at 6000× *g* for 30 min and then filtered through a 0.45 μm filter. The medium (MVB) was kept frozen until use. The controls were cultivated under identical conditions without inoculation of *B. subtilis* MV1.

#### 2.4.2. Biotransformation of *M. verticillata* Leaves by *Aspergillus oryzae*

*Aspergillus oryzae* KCTC 6983 was purchased from the Korean Collection for Type Cultures (KCTC) in the Korea Research Institute of Bioscience and Biotechnology (Daejeon, Korea) and grown at 25 °C on a malt extract agar or a YPD agar plate. One gram of *M. verticillata* leaves was added to 200 mL of MEB, sonicated for 60 min, and then, 2 mL of the *A. oryzae* spores (OD_620 nm_ = 1) was inoculated into the broth. Cultivation was carried out at 30 °C. Following cultivation for 7 days, the medium was centrifuged at 6000× *g* for 30 min and then filtered through a 0.45 μm filter. The medium (MVA) was kept frozen until use. The controls were cultivated under identical conditions without inoculation of *A. oryzae*.

#### 2.4.3. Biotransformation of Aqueous Extracts of *M. verticillata* Leaves by *B. subtilis* MV1

Four hundred milligrams of the MVW was added to 200 mL of LB broth, sonicated for 60 min, and then 2 mL of *B. subtilis* MV1 (OD_600 nm_ = 1) was inoculated into the broth. Cultivation was carried out at 30 °C. Following cultivation for 7 days, the medium was separated by centrifugation at 6000× *g* for 30 min and then filtered through a 0.45 μm filter. The medium (MVWB) was kept frozen until use.

### 2.5. Determination of Phenolic and Flavonoid Contents

Total phenolic content (TPC) was determined, as described previously [19]. Gallic acid was used as a standard, and the TPC was expressed as mg of gallic acid equivalent (GAE) per gram. Total flavonoid content (TFC) was measured as described previously [19]. Quercetin was used as a standard and the TFC was expressed as mg of quercetin equivalent (QE) per gram.

### 2.6. Antioxidant Activity Assay

DPPH free radical scavenging ability and ferric reducing antioxidant power (FRAP) assay were determined as described previously by Moon et al. [19]. DPPH value was compared with a quercetin and expressed as mg of quercetin per gram. FRAP value was compared with a FeSO_4_ standard curve and expressed as mg of FeSO_4_ per gram.

### 2.7. Cell Culture and Cell Viability Assay

Murine C3H10T1/2 MSC line was purchased from the American Type Culture Collection (Rockville, MD, USA). C3H10T1/2 MSCs were cultured in MEM-α with 10% heat-inactivated FBS and 1% antibiotics (penicillin 10,000 U/mL and streptomycin 10,000 mg/mL) and maintained at 37 °C under 5% CO_2_ in a humidified culture chamber. For the cell viability assay, C3H10T1/2 cells (3 × 10^3^ cells/well) were seeded into 96-well plates and cultured for 24 h. The cells were then treated with *M. verticillata* leaf extracts (MVW, MVB) at various concentrations ranging from 1 to 100 μg/mL and then cultured for 1–3 days. The media was replaced with fresh media (100 μL/well) containing 0.5 mg/mL MTT solution, and the cells were incubated at 37 °C for 1 h. The formazan crystals that were converted from MTT during the reaction of oxidoreductase in the living cells, were dissolved in 100 μL of DMSO, and their absorbance was measured at 570 nm using a microplate reader.

### 2.8. Inhibition Activity of Osteoclast Differentiation

RAW264.7 cells at a density of 1 × 10^5^ cells/mL in 12-well plates were cultured in DMEM supplemented with 10% FBS and 1% antibiotics at 37 °C in a 5% CO_2_ incubator. Following incubation for 24 h, the cells were stimulated with 50 ng/mL RANKL with or without the *M. verticillata* aqueous extracts at a concentration of 100 μg /mL and then incubated further, for an additional 6 days. The medium was replaced every 2 days. Following incubation and treatment for 6 days, the cells were observed using an inverted microscope (Eclipse TS100, Nikon, Tokyo, Japan).

### 2.9. Alkaline Phosphatase (ALP) Activity Assay and ALP Staining

The ALP activity assay involved the following C3H10T1/2 cells (4 × 10^4^ cells/well) were seeded in 12-well plates. After 24 h, the media was replaced with osteogenic media, MEM-α was supplemented with 10% heat-inactivated FBS, antibiotics, 50 μg/mL ascorbic acid, and 10 mM β-glycerolphosphate, with or without *M. verticillata* leaf extract (range 1 to 50 μg/mL), and the media was replaced every 3 days. The supernatant of *B. subtilis* MV1 and BMP-2 was used as negative control and positive control, respectively. Following 9 days, the cells were rinsed with phosphate-buffered saline (PBS, pH 7.4) three times and then harvested. The cell lysis buffer was added, maintained for 10 min at room temperature, and centrifuged at 16,000 *g* for 1 min. The protein concentration of the supernatant was measured by Bradford assay using bovine serum albumin (BSA) as a standard. Fifty microliters of each lysate were transferred to 96-well plate, 100 μL of *para*-nitro phenyl phosphate (*p*-NPP, Thermo Fisher Scientific) was added to each well and then incubated for 1 h at room temperature. The released *p*-NP was determined at 405 nm. ALP activity was calculated using the following equation.

ALP activity (nM/μg/min) = released *p*-NP/protein concentration of cell extract × microliters of cell extract/min.

For ALP staining, the cells (3 × 10^3^ cells/well) were seeded into a 96-well plate and cultured using the same method as the ALP activity assay. Following culturing for 9 days, the cells were rinsed with PBS three times, and the BCIP/NBT substrate (Promega, USA) was added into each well. The reaction was stopped after 2 h, with 5 mM EDTA (pH 8.0). Image J software (version 1.52a, National Institutes of Health, Bethesda, MD, USA) was used to quantify the area of mineralized nodules. MVW was incubated at 121 °C for 15 min to test the thermal stability.

### 2.10. Mineralized Nodule Measurement

Mineralization of C3H10T1/2 cells was measured according to the method described by Song et al. [20]. Alizarin Red S was used to measure the mineralization of cells as it was selectively bound to calcium and then stained it either red or orange. Cells (4 × 10^3^ cells/well) were seeded into a 96-well plate and cultured with osteogenic medium with or without the *M. verticillata* aqueous extracts. The medium was changed every 2 days. Following incubation for 21 days, the cells were washed with PBS, fixed with 10% buffered formaldehyde for 15 min, rinsed with distilled water two times, and stained with 2% Alizarin Red S (pH 4.2) for 30 min at room temperature. After staining, the cells were washed with distilled water on a shaking platform for 5 min; this washing step was repeated 5 times. Images of the mineralized nodules were photographed using a microscope.

### 2.11. Western Blotting

The western blot assay involved the following; C3H10T1/2 cells were cultured in 6 well plates for 2 days. The cells were treated with MVB during incubation. For inhibition of the extracellular signal-regulated kinase (ERK), p38, or the transforming growth factor-β (TGF-β) receptor, the cells were pretreated with 10 µM ERK inhibitor (U0126), p38 inhibitor (SB203580), TGF-β receptor inhibitor (SB525334), respectively, for 1 h followed by MVB treatment for 1 h [21]. The cells were rinsed with PBS three times, harvested, and lysed in radioimmunoprecipitation assay (RIPA) buffer. Protein lysate was separated by sodium dodecyl sulfate-polyacrylamide gel electrophoresis (SDS-PAGE) and then transferred to nitrocellulose membranes. The transfer membrane was blocked with 5% (*w*/*v*) BSA for 1 h and washed three times for 15 min with PBS supplemented with 0.1% Triton X-100 (PBST). The primary antibody against ERK (1: 200, Santa Cruz Biotechnology, Dallas, TX, USA), p-ERK (1:200, Santa Cruz Biotechnology), p38 (1:1000, Cell Signaling Technology, Danvers, MA, USA), p-p38 (1:1000, Cell Signaling Technology), and β-actin (1: 200, Santa Cruz Biotechnology) was added to the transfer membrane and incubated for 20 h and then washed with PBST. HRP-conjugated secondary antibody (1:2000, Sigma-Aldrich) was added to the transfer membrane and incubated for 1 h and washed with PBST. Then chemiluminescent reagents (Luminata Forte Western HRP substrate; Merck Millipore, Billerica, MA, USA) were added to the transfer membrane, and the bands were detected using a ChemiDoc^TM^ XRS+ imaging system (Biorad, Hercules, CA, USA).

### 2.12. Total RNA Isolation and RT-PCR

For measurement of mRNA expression in C3H10T1/2 cells, total RNA was isolated from the differentiated cells using AccuPrep^®^ Universal RNA Extraction Kit (Bioneer, Daejeon, Korea), and the RNA concentration was determined using a Thermo Fisher Scientific NanoDrop2000. RNase-free DNase I (Thermo Fisher Scientific) was added to the RNA to remove genomic DNA contamination according to the manufacturer’s instructions. Complementary DNA (cDNA) was synthesized from 0.5 μg of the total RNA using a RevertAid First Strand cDNA Synthesis Kit (Thermo Fisher Scientific) with oligo-dT. Real-time polymerase chain reaction (RT-PCR) was performed with a StepOnePlus Real-Time PCR system (Applied Biosystems, Foster City, CA, USA) using SYBR^®^ Premix Ex Taq kit (Takara Bio Inc., Shiga, Japan) according to the manufacturer’s protocol. The PCR assay protocol was 40 cycles at 94, 57, and 72 °C for 5, 30, and 30 s, respectively. Data were presented as relative mRNA level of the gene of interest normalized to the mRNA level of the endogenous GAPDH. Relative target gene expression was quantified using the comparative CT method. Primer sequences are listed in Table 1.

### 2.13. Liquid Chromatography and Mass Spectrometry Analysis

The aqueous extracts of *M. verticillata* leaves were analyzed via ultra-performance liquid chromatography and a quadrupole-time-of-flight mass spectrometry (UPLC-Q-TOF MS) system. The aqueous extracts (30 μL) of MVW and MVWB were injected into a Waters ACQUITY I-Class UPLC system (Waters, Milford, MA, USA) and an ACQUITY UPLC BEH C_18_ column (2.1 × 100 mm, 1.7 μm; Waters) was applied for the analyses. The UPLC condition for the component analysis of the MVW and MVWB was performed according to the methods described by Bao et al. with minor modifications [7]. The elution solvents were (A) aqueous 0.1% formic acid in water and (B) 0.1% formic acid in acetonitrile. The solvent 0.1% formic acid in acetonitrile was used for all of the following sample elutions, 1% for 0–2 min, followed by a linear gradient from 1% to 80% in the first 2–10 min, a linear gradient from 80% to 100% from 10 to 10.5 min, 100% for 10.5–12 min, 100% to 1% from 12 to 13 min, and 1% from 13 to 15 min. The Q-TOF mass spectrometer (maXis HDTM, Bruker, Billerica, MA, USA) was operated in negative mode with a capillary voltage of 4500 V and an end-plate offset of −500 V. The scanning mass range (*m*/*z*) was from 50 to 1000, and the collision energy was 23 eV. Nitrogen was used as the nebulizing gas at a flow rate of 8 L/min, a temperature of 200 °C, and a pressure of 1.0 bar. Bruker Compass Data Analysis 4.2 (Bruker) was used to coordinate the LC-MS system.

### 2.14. Statistical Analysis

All experiments were performed in triplicate. Data were analyzed using SigmaPlot software (version 12.5; Systat, San Jose, CA, USA) and expressed as the mean ± standard deviation. The data were analyzed by the one-way analysis of variance test, and the mean value was considered to be significantly different at *p* < 0.05, *p* < 0.01, and *p* < 0.001.

## 3. Results

### 3.1. Methanol and Aqueous Extracts of M. verticillata Leaves Exhibit Osteoblast Differentiation

**Figure 1:**
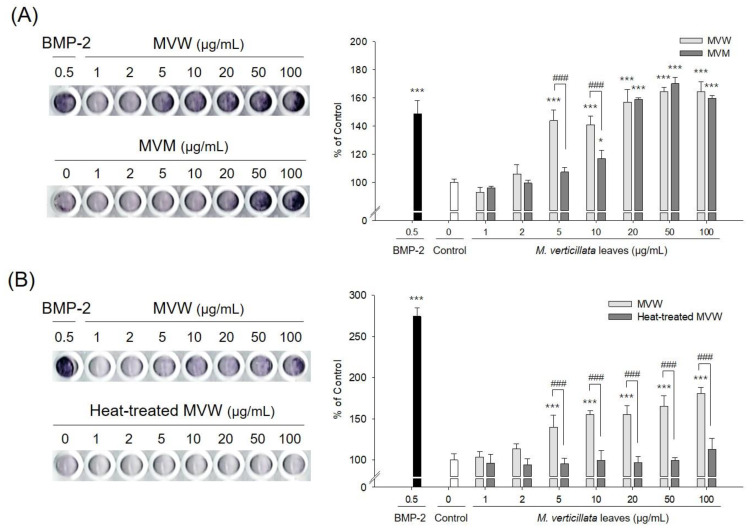
Effect of the extraction solvents (**A**) and heat treatments (**B**) on osteoblast differentiation of *M. verticillata* leaves. MVW: aqueous extracts of *M. verticillata* leaves; MVM: methanol extracts of *M. verticillata* leaves; BMP-2: bone morphogenetic protein-2. Significant differences were observed when compared with the controls: * *p* < 0.05, *** *p* < 0.001; significant differences were also observed between the two groups: ^###^ *p* < 0.001.

The aqueous and methanol extracts of the *M. verticillata* leaves were compared to the affected osteoblast differentiation according to the type of extraction solvent used. Both MVW and MVM exhibited ALP activity, which was a critical marker for osteoblast differentiation. However, MVM showed significant activity above a concentration of 20 μg/mL, while MVW showed activity over 5 μg/mL, which indicated that water was a more optimal extractant than methanol (Figure 1A). The ALP activity of MVW was examined via heat treatment. The thermal stability of MVW involved the following; MVW was treated at 121 °C for 15 min. The ALP activity of MVW was completely lost during the heat treatment, which indicated that the components of MVW had affected the osteoblast differentiation, which was not heat stable (Figure 1B). MVW was also examined to determine whether it exhibited inhibitory activity against osteoclast differentiation. RANKL was added to RAW264.7 cells to induce multinucleated cells in the presence or absence of MVW. The results found that MVW did not inhibit osteoclast differentiation (Figure S1).

### 3.2. Biotransformation of the M. verticillata Leaves by B. subtilis MV1 Enhances the Phenolic and Flavonoid Compounds and the Antioxidant Activities

The *M. verticillata* leaves possess abundant phenolic compounds and antioxidant activity [7,24]. Many types of phenolic compounds and flavonoids exhibit antioxidant activity, which is involved in bone formation by reducing oxidative stress, and inhibiting NF-κB activation [25]. To enhance the antioxidant activity of the *M. verticillata* leaves, the leaves were fermented with microorganisms. Two bacteria were isolated from the *M. verticillata* leaves, and the 16s rRNA regions of the bacteria were amplified and sequenced. The sequences obtained were analyzed using the online BLASTN alignment tool, which compared them with sequences in GenBank. The 16s rDNA gene sequences of isolate 1 revealed that the strain showed 100% similarity to that of the *Bacillus subtilis* strain DSM 10 (accession no. NR_027552), *B. subtilis* strain NBRC 13719 (accession no. NR_112629), and *B. subtilis* strain JCM 1465 (accession no. NR_113265). Therefore, the strain was termed *B. subtilis* MV1. The 16s rDNA gene sequences of isolate 2 revealed that the strain showed 99.93% similarity to that of the *Bacillus velezensis* strain BvL03 (accession no. CP041192.1), *B. velezensis* strain AL7 (accession no. CP045926.1), and *B. velezensis* FJAT-52631 (accession no. CP045186.1). Therefore, this strain was termed *B. velezensis* MV2. Among these strains, the *M. verticillata* leaves were fermented with *B. subtilis* MV1, because *B. subtilis* is generally recognized as safe (GRAS). MVW was also fermented with *B. subtilis* MV1 to exclude whether the increase in antioxidant activity by *B. subtilis* MV1 was caused by the increase in extraction yield of TPC and TFC. In addition to the *Bacillus*, *A. oryzae* was also widely used for bioconversion. Therefore, whether the antioxidant activity of the *M. vertillata* leaves was enhanced by *A. oryzae* fermentation was tested. The effects of the MVW, MVB, MVWB, and MVA fermentation on the TPC, TFC, and the antioxidant activities were measured (Table 2). TPC was measured using the Folin-Ciocalteu method. The data revealed that the TPC of MVW, MVB, MVWB, and MVA were 8.6 ± 0.4, 23.6 ± 0.2, 10.6 ± 0.4, and 8.0 ± 0.8 mg GAE/g dry weight, respectively. The TFC of MVW, MVB, MVWB, and MVA were 2.4 ± 0.1, 13.2 ± 0.3, 3.0 ± 0.2 and 1.9 ± 0.1 mg QE/g dry weight, respectively. The TPC and TFC of MVB were increased by about 2.7-fold and 5.5-fold compared with those of MVW, respectively. The antioxidant activities of MVW, MVB, MVWB, and MVA were determined by DPPH and FRAP. The DPPH radical scavenging activities of MVW, MVB, MVWB, and MVA were 1.9 ± 0.2, 7.5 ± 0.4, 4.1 ± 0.3, and 1.4 ± 0.2 mg QE/g, respectively. The FRAP activities of MVW, MVB, MVWB, and MVA were 12.3 ± 0.1, 74.5 ± 1.1, 39.7 ± 0.1, and 6.2 ± 0.1 mg FeSO_4_/g, respectively. In addition to increasing TPC and TFC of MVB, the antioxidant activities of MVB were increased by about 3.9-fold and 6.1-fold compared with those of MVW, respectively. In contrast to MVB, the TPC and TFC of MVA were decreased slightly, and the antioxidant activities of MVA were also decreased compared to MVW. This indicated that the increase in TPC and TFC was proportional to the antioxidant activity. The TPC and TFC of MVWB were increased by about 1.2-fold compared with those of the MVW, while DPPH and FRAP of MVWB were increased by about 2.2-fold and 3.2-fold, respectively, compared with those of MVW. These results suggested that the components of MVW were converted to other components during *B. subtilis* MV1 fermentation.

To determine the components of aqueous extracts of the *M. verticillata* leaves that affect antioxidant activities, MVW and MVWB were analyzed by LC-MS. In LC-MS analysis, two compounds, kaempferol and kaempferol-glucuronide, were identified (Table S1). Kaempferol of MVWB was increased by about 3.2-fold, but kaempferol-glucuronide of MVWB was decreased by about 0.54-fold compared to MVW.

### 3.3. Biotransformation of M. verticillata Leaves by the Genus Bacillus Enhances the Osteoblast Differentiation of C3H10T1/2 Cells and the Osteoblast Differentiation Occurs via the TGF-β Signaling Pathway

The *M. verticillata* leaves were fermented with *B. subtilis* MV1 and *B. velezensis* MV2 to examine the ALP activity of the C3H10T1/2 MSCs. The leaves were also fermented with *B. subtilis* strain 168 to confirm whether the change in osteoblast differentiation by the *M. verticillata* leaves was characteristic of the strains isolated from the *M. verticillata* leaves. The genus *Bacillus* used in this study exhibited the ability to enhance osteoblast differentiation of the *M. verticillata* leaves. Among them, *B. subtilis* MV1 was used for further study because it exhibited greater efficacy at improving ALP activity than the other *Bacillus* spp. In addition to the *Bacillus* spp., *M. verticillata* leaves were also fermented with *A. oryzae* (Figure S2). Unlike the results for the genus *Bacillus*, the ALP activity of the *M. verticillata* leaves fermented with *A. oryzae* had decreased following fermentation.

**Figure 2:**
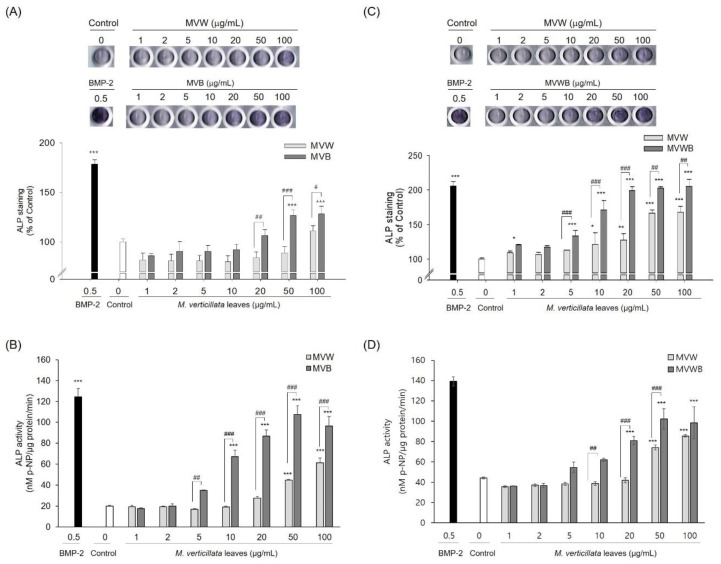
Effects of MVW, MVB, and MVWB on the alkaline phosphatase (ALP) activity of C3H10T1/2 cells. Visualization of the osteoblast differentiation of C3H10T1/2 cells by MVW and MVB (**A**) and MVW and MVWB (**C**). ALP activity of C3H10T1/2 cells by MVW and MVB (**B**) and MVW and MVWB (**D**). MVWB: the fermented MVW by *B. subtilis* MV1. Significant differences were observed when compared with the controls: * *p* < 0.05, ** *p* < 0.01, *** *p* < 0.001; significant differences between MVW and MVB or MVWB were observed: ^#^ *p* < 0.05, ^##^ *p* < 0.01, ^###^ *p* < 0.001.

Prior to testing osteoblast differentiation of the *M. verticillata* leaves, MVW and MVB were tested for cytotoxicity against C3H10T1/2 cells by MTT assay. MVW and MVB exhibited no significant cytotoxic effects in C3H10T1/2 cells at concentrations of up to 100 μg/mL (Figure S3). The effect of MVW, MVB, and MVWB on osteoblast differentiation of C3H10T1/2 cells was examined via ALP activity. ALP staining of C3H10T1/2 cells was higher in MVB and MVWB than in MVW (Figure 2A,C). ALP activity was also determined by using *p*-NPP substrate (Figure 2B,D). ALP activity was also higher in MVB and MVWB than in MVW. The ALP activity of the cells treated with MVW, MVB, and MVWB began to increase at a concentration of 20, 5, and 10 μg/mL, respectively, indicating that osteoblast differentiation of the *M. verticillata* leaves was improved by the *B. subtilis* MV1. The ALP activity of MVB and MVWB were increased by about 2 to 3-fold compared with that of MVW, and the increasing rate of ALP activity by MVB was higher than that of MVWB. In particular, the higher ALP activity observed in MVWB compared to that of MVW helped reinforce the fact that the increase in ALP activity was not related to the extraction yield of the biologically active compounds in the *M. verticillata* leaves. These results indicated that fermentation by *B. subtilis* MV1 increased the extraction yield of the biologically active compounds, as well as the osteoblast differentiation of the biologically active compounds in the *M. verticillata* leaves.

MVW, MVB, and MVWB also induced intracellular mineralization during osteogenesis in C3H10T1/2 cells (Figure S4). Mineralization in the C3H10T1/2 cells had occurred after 21 days in culture. The intracellular mineralization of C3H10T1/2 cells was detected by Alizarin Red S staining, which binds to calcium to form a pigment that appears orange to red. The cells treated without the addition of *M. verticillata* extract were not stained, but the cells treated with MVW, MVB, MVWB, and the positive control BMP-2 were all stained red. Similar to the results of the ALP activity, cells treated with MVB and MVWB were stained red in a larger area than cells treated with MVW. This indicated that a significant amount of calcium precipitation occurred in the cells treated with MVB and MVWB.

**Figure 3:**
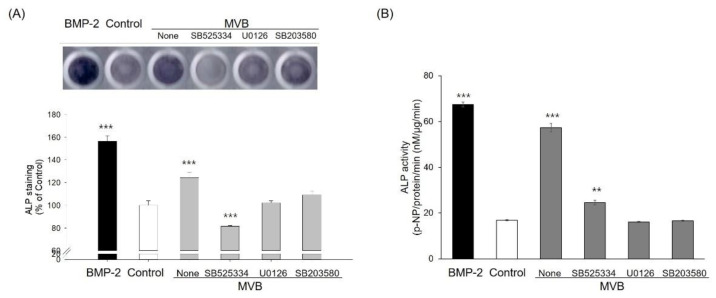
Effect of TGF-β signaling inhibitors on osteoblast differentiation. ALP staining (**A**) and ALP activity (**B**) of C3H10T1/2 cells by MVB and TGF-β signaling inhibitors. Significant differences were observed when compared with the controls: ** *p* < 0.01, *** *p* < 0.001.

**Figure 4:**
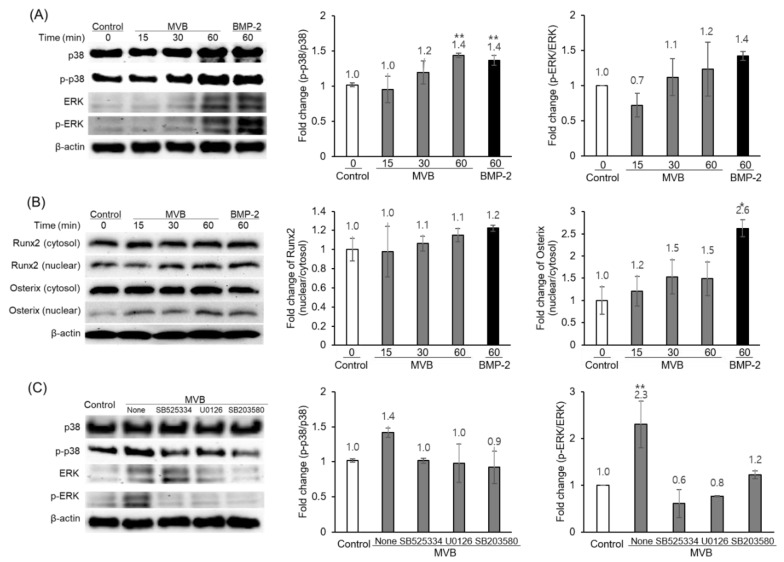
Western blot showing activation of transforming growth factor-β (TGF-β) signaling pathway by MVB treatment in C3H10T1/2 cells. (**A**) Time-course analysis of the non-canonical signaling of TGF-β pathway after treatment with 100 μg/mL MVB. (**B**) Time-course analysis of Runx2 and Osterix after treatment with 100 μg/mL MVB. (**C**) Pre-treating cells with inhibitors of TGF-β signaling pathway attenuated MVB (100 μg/mL)-induced p38 and ERK activation. Significant difference compared with control: * *p* < 0.05, ** *p* < 0.01.

**Figure 5:**
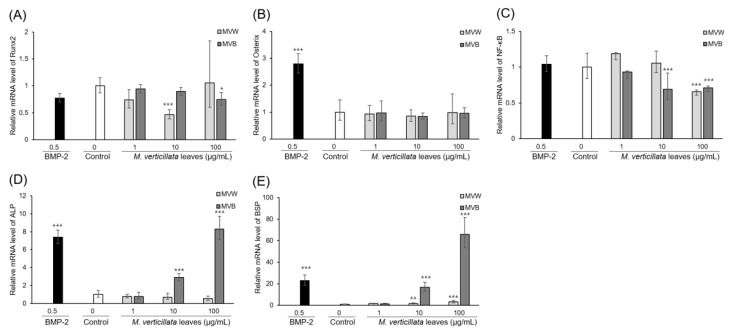
Effects of MVW and MVB on gene expression related to osteoblast differentiation in C3H10T1/2 cells. C3H10T1/2 cells were treated with MVW and MVB, cultured for 9 days, and harvested. Total RNA was isolated from the cells, and the cDNA was synthesized using the total RNA as a template. The mRNA expression of marker genes for osteoblast differentiation was measured by RT-PCR. The osteogenic markers are (**A**) Runx2, (**B**) Osterix, (**C**) NF-κB, (**D**) ALP, (**E**) BSP. Significant differences compared with the controls: * *p* < 0.05, ** *p* < 0.01, *** *p* < 0.001.

C3H10T1/2 cells are known to differentiate into osteoblasts, chondrocytes, and adipocytes in response to certain growth factors [26]. In order to identify the regulatory effects of the aqueous extracts of the *M. verticillata* leaves on the MSCs differentiation into osteoblasts, C3H10T1/2 cells were treated with MVB and TGF-β signaling inhibitors at a concentration of 100 μg/mL and 10 μM, respectively. As depicted in Figure 3, the SB525334, U0126, and SB203580 inhibitors of the TGF-β1 receptor, MEK1 and MEK2, and p38 MAP kinase, respectively, had attenuated the ALP activity of C3H10T1/2 cells with MVB. Furthermore, phosphorylation of p38 and ERK by MVB was increased in a time-dependent manner (Figure 4A). In addition, the Runx2 and Osterix in the nucleus were increased in a time-dependent manner (Figure 4B). Likewise, the result of ALP activity, phosphorylation of p38 and ERK, was also attenuated by the TGF-β signaling inhibitors (Figure 4C). This result implied that the MVB stimulates MSCs differentiation into osteoblast through TGF-β signaling. The mRNA expression of various genes related to the MSCs differentiation into osteoblasts was also measured by RT-PCR (Figure 5). The mRNA expression of Runx2 and Osterix, the major transcriptional regulators of osteoblast differentiation, were not affected by MVW and MVB. However, the mRNA expression of ALP and BSP, the major osteoblast differentiation markers, in the cells treated with MVB had increased in a dose-dependent manner. The mRNA expression level of ALP and BSP in cells treated with 100 μg/mL of MVB were increased by about 9.4 and 65-fold, respectively, compared to the non-treated cells. However, the mRNA expression of ALP and BSP in cells treated with MVW was slightly increased compared to the cells treated with MVB. This indicated that MVB stimulated MSCs differentiation into osteoblasts at a more optimal rate than the MVW. NF-κB, a negative regulator of osteoblasts, was known to inhibit the expression of matrix proteins by directly interacting with the promoter regions of these genes or by inhibiting the binding of Runx2 and β-catenin to the promoter regions [27]. The mRNA expression of NF-κB in the cells treated with 100 μg/mL of MVW and MVB was decreased by about 30% compared to that of the controls. This suggested that MSCs differentiation into osteoblasts by MVW and MVB was affected by the negative regulation of NF-κB.

## 4. Discussion

This study investigated whether antioxidant and osteoblast differentiation of *M. verticillata* leaves could be improved through microbial fermentation and how the substances that affect this activity are converted. We examined the antioxidant activity and osteoblast differentiation for aqueous extracts of the *M. verticillata* leaves. LC-MS analysis was also performed to define the substances converted by fermentation.

Both the methanol and aqueous extracts of the *M. verticillata* leaves showed ALP activity, and the aqueous extracts exhibited stronger activity than the methanol extracts. Recently, Shim et al. reported that a water extract of *M. verticillata* seeds showed inhibitory activity of RANKL-induced osteoclast differentiation [5]. The current study tested whether aqueous extracts of *M. verticillata* leaves exhibited inhibitory activity against osteoclast differentiation; however, the aqueous extracts were not found to exhibit inhibitory activity. In many cases, the substances that induced osteogenesis or inhibited osteoclastogenesis were soluble in organic solvents such as methanol or ethanol and insoluble in water. Water solubility was highly correlated with oral bioavailability. Furthermore, solubility in water increased the bioavailability and was found to be more useful than the organic solvents [28,29]. In this respect, the bioavailability of the aqueous extracts of *M. verticillata* leaves might be advantageous because of their high solubility in water.

*M. verticillata* leaves were fermented with *B. subtilis* MV1 to increase the extraction yield of TPC and TFC, affecting the antioxidant activity and biological activity of the biologically active compounds derived from the *M. verticillata* leaves. The aqueous extract (MVW) was also fermented with *B. subtilis* MV1 to exclude the effect of the increased extraction yield of the biologically active compounds from the leaves during fermentation. The TPC and TFC of MVWB were not significantly different compared with MVW, but the antioxidant activities were increased by about 2 to 3-fold. This indicated that biotransformation with the *B. subtilis* MV1 influenced the biological activity of the aqueous extracts of the *M. verticillata* leaves by enzymatic conversion of the biologically active compounds. The ALP activity and bone mineralization in C3H10T1/2 cells treated with the fermented extracts of *M. verticillata* leaves were examined as an indicator of osteoblast differentiation. The aqueous extracts of the *M. verticillata* leaves fermented by *B. subtilis* MV1 exhibited higher ALP and bone mineralization activity than the unfermented extracts.

Attempts at biotransformation using the genus *Bacillus* and *A. oryzae* indicated that *B. subtilis* MV1 promoted osteoblast differentiation, while *A. oryzae* reduced osteoblast differentiation. These results suggested that changes in the biological activity of the phytochemicals by microbial fermentation may vary depending on the type of the microorganism used. The increase of phenolic compound extraction yield as well as osteoblast differentiation of the aqueous extracts of the *M. verticillata* leaves via *B. subtilis* MV1 fermentation were similar to the results of bioconversion using other microorganisms. Dajanta et al. reported that the phenolic contents of black and yellow soybeans fermented with *B. subtilis* were increased by about 9-fold and 3-fold, respectively, compared with that of the non-fermented soybeans, [18]. Razak et al. also reported that rice bran fermented with *A. oryzae* and *R. oryzae* showed higher phenolic and organic acid contents and higher antioxidant, tyrosinase, and elastase inhibition activities than the non-fermented rice bran [13]. Seong et al. also reported that *Lespedeza cuneata* extracts fermented with *Lactobacillus pentosus* showed increased antiaging activities compared with the non-fermented extract, ultimately six compounds, including isovitexin, avicularin, quercitrin, juglanin, quercetin, and kaempferol, were converted by fermentation [12].

Various pathways, including TGF-β signaling, Wnt signaling, and Hedgehog signaling, contributed to differentiation from the MSCs to the osteoblasts (Figure S5) [30]. The western blotting assay and mRNA expression of the various pathway genes associated with osteoblast differentiation were measured to investigate the regulatory effects of the aqueous extracts of the *M. verticillata* leaves on osteoblast differentiation. The phytochemicals that exhibited osteoblast differentiation were primarily heat-labile phenolic compounds and terpenes and were known to promote osteoblast differentiation through stimulation of the various pathways. Since osteoblast differentiation of the aqueous extracts from the *M. verticillata* leaves was lost by heat treatment, the main substances of the extracts are considered to be phenolic compounds. However, other compounds were not identified except for kaempferol and kaempferol derivative. Various studies have been conducted, including the composition and biological activities of *M. verticillata* seeds, but few have been studied on *M. verticillata* leaves. Bao et al. reported the components of ethanol extracts of *M. verticillata* leaves, stems and seeds. In *M. verticillata* leaves, various phenolic compounds including myricetin, catechin, quercetin, kaempferol, and their derivatives were identified [7]. The inhibitors of TGF-β signaling attenuated the MVB-induced phosphorylation of p38 and ERK as well as the MVB-induced osteoblast differentiation. Epicatechin gallate, which is a derivative of catechin, was known to stimulate osteoblast differentiation via Runx2 and a transcriptional coactivator with PCZ-binding motif-mediated transcriptional activation [31]. Myricetin is also known to induce osteoblast differentiation through the BMP-2/p38 MAP kinase pathway [32]. Quercetin stimulates osteoblast differentiation through ERK and the estrogen receptor pathway, and kaempferol stimulates osteoblast differentiation through the ERK, estrogen receptor, and Wnt signaling pathways [33,34].

To the best of our knowledge, this study was the first to provide evidence that the aqueous extracts of *M. verticillata* leaves exhibited an osteogenic effect and that this effect was enhanced by fermentation with *B. subtilis* MV1. In addition, the aqueous extracts of *M. verticillata* leaves might be useful as a pharmacological agent for osteoporosis because the aqueous extracts are advantageous for use due to their high solubility in water. However, identification of the specific biologically active compounds that affected osteoblast differentiation and the regulatory pathways of how these compounds relate to osteoblast differentiation still requires further investigation.

## Figures and Tables

**Table 1:** RT-PCR primers used in this study.

Target Gene	Sequence (5’→3’)	Product		
		Orientation	Size (Base Pairs)	Reference
GAPDH	ACCACAGTCCATGCCATCAC	Sense	452	[22]
	TCCACCACCCTGTTGCTGTA	Antisense		
Runx2	AAGCTGCGGCAAGACAAG	Sense	61	[23]
	TCAAATCTGCAGCTTCAAGG	Antisense		
Osterix	GCTAGAGATCTGAGCCGGGTA	Sense	56	[23]
	AAGAGACCTGGCAAGAGG	Antisense		
ALP	AAACCCAGAACACAAGCATTCC	Sense	218	[23]
	TCCACCAGCAAGAAGAAGCC	Antisense		
BSP	TTGAGTTAGCGGCACTCCAA	Sense	78	This study
	CGTCGCTTTCCTTCACTTTT	Antisense		
NF-kB	ACACGAGGCTACAACTCTGC	Sense	164	This study
	GGTACCCCCAGAGACCTCAT	Antisense		

**Table 2:** Total phenolic content (TPC), total flavonoid content (TFC), and antioxidant activities of the aqueous extract (MVW), the aqueous extract of fermented leaves by *B. subtilis* MV1 (MVB), the fermented MVW by *B. subtilis* MV1 (MVWB), and aqueous extract of fermented leaves by *A. oryzae* (MVA).

Sample	TPC (mg GAE/g)	TFC (mg QE/g)	DPPH (mg QE/g)	FRAP (mg FeSO_4_/g)
MVW	8.6 ± 0.4	2.4 ± 0.1	1.9 ± 0.2	12.3 ± 0.1
MVB	23.6 ± 0.2 ***	13.2 ± 0.3 ***	7.5 ± 0.4 ***	74.5 ± 1.1 ***
MVWB	10.6 ± 0.4 ***	3.0 ± 0.2	4.1 ± 0.3 ***	39.7 ± 0.1 ***
MVA	8 ± 0.8	1.9 ± 0.1	1.4 ± 0.2	6.2 ± 0.1 ***

Data are expressed as mean ± standard deviation (n = 3). Significant differences were observed when compared with MVW: *** *p* < 0.001. TPC, TFC, and antioxidant activities of MVB and MVWB were carried out after 7 days of fermentation.

## Supplementary Materials

The following are available online at https://www.mdpi.com/2304-8158/9/5/671/s1. Figure S1: Effect of aqueous extract of *M. verticillata* leaves on osteoclast differentiation. Figure S2: Effect of microbial fermentation of *M. verticillata* leaves on osteoblast differentiation. Figure S3: Effect of MVW and MVB on the viability of C3H10T1/2 cells. Figure S4: Effect of MVW, MVB, and MVWB on the mineralization of C3H10T1/2 cells using Alizarin Red S dye. Figure S5: Signaling pathways for osteoblast differentiation. Table S1: Compounds detected by LC-MS analysis of the aqueous extracts of the *M. verticillata* leaves.
