# Supplementary material for: Bacillus subtilis Fermentation of Malva verticillata Leaves Enhances Antioxidant Activity and Osteoblast Differentiation
Source: Foods. 2020 May 22;9(5):671. doi: 10.3390/foods9050671 (PMC7278731; doi:10.3390/foods9050671)
Supplement: Supplementary file 1 [file foods-09-00671-s001.pdf]

Supplementary data

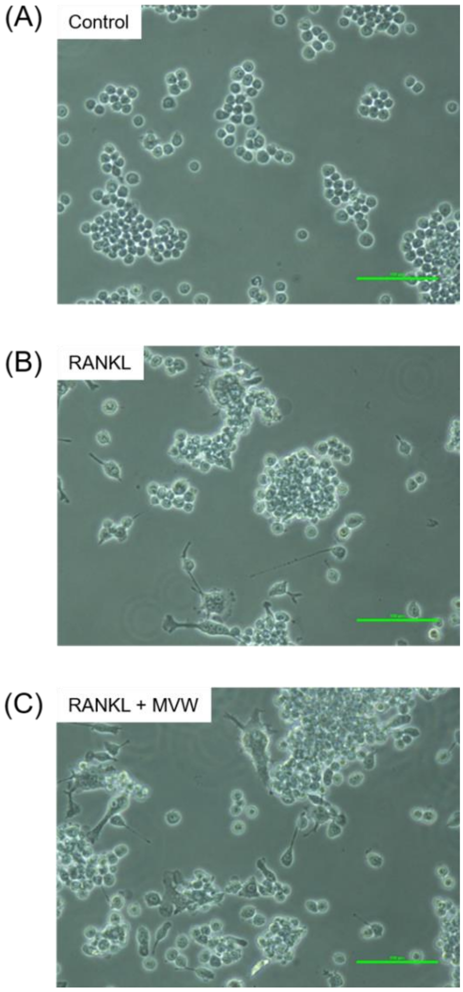

Supplementary Figure 1. Effect of aqueous extract of *M. verticillata* leaves on osteoclast differentiation.

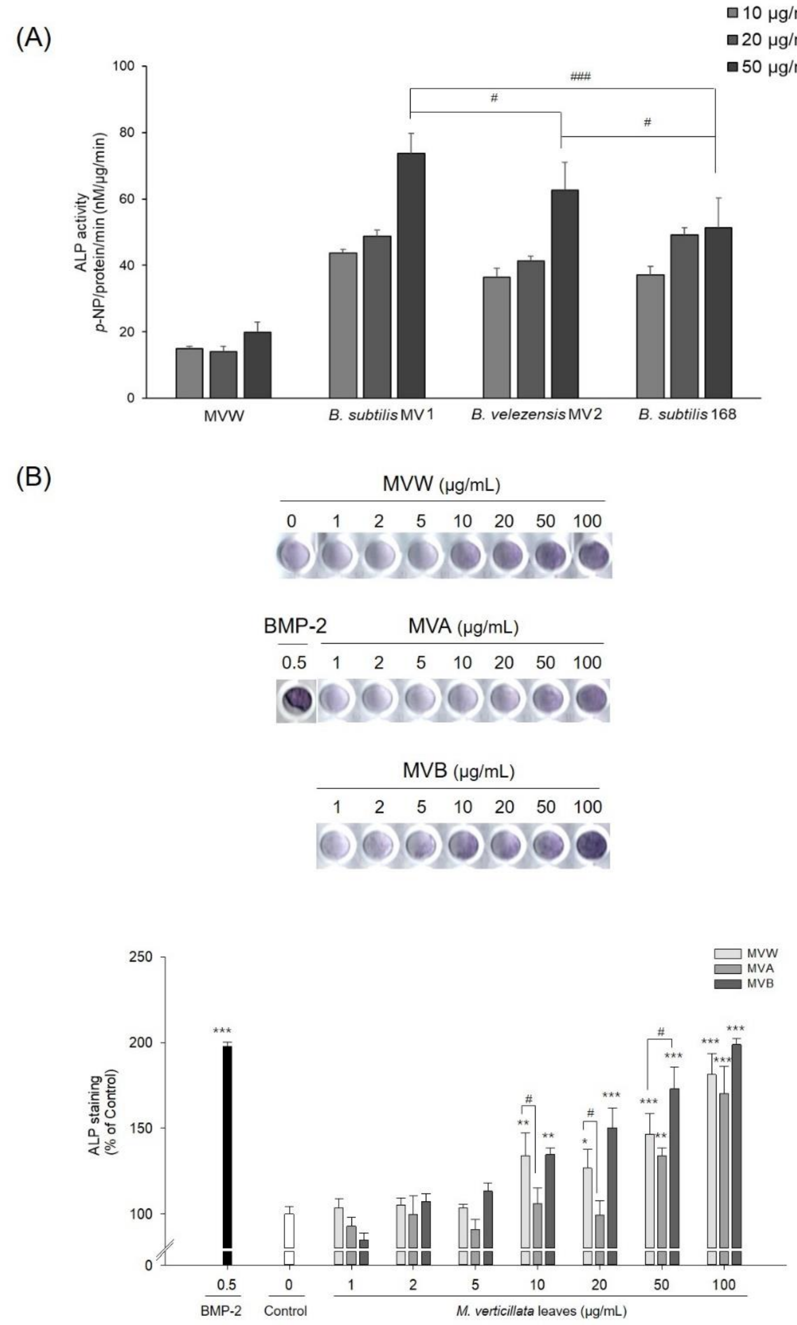

Supplementary Figure 2. Effect of microbial fermentation of *M. verticillata* leaves on osteoblast differentiation. (A) The *M. verticillata* leaves were fermented with *B. subtilis* MV1, *B. velezensis* MV2 and *B. subtilis* 168, respectively, at 30 °C for 7 days. The supernatant was used as aqueous extracts of the fermented leaves. C3H10T1/2 cells were treated with the extracts and cultured for 9 days. The cell extracts were reacted with *p*-NPP substrate for 1 h and the released *p*-NP was determined at 405 nm. (B) The *M. verticillata* leaves were fermented with *A. oryzae* or *B. subtilis* MV1 for 7 days. The supernatant was used as aqueous extracts of the fermented leaves. C3H10T1/2 cells were treated with the extracts and cultured for 9 days. The cells were stained with BCIP/NBT substrate to detect ALP activity. Significant differences compared with the controls: $^{*}p < 0.05$, $^{**}\ p < 0.01$, $^{***}\ p < 0.001$; significant differences between the two groups: $^{\#}\ p < 0.05$, $^{\#\#\#}\ p < 0.001$.

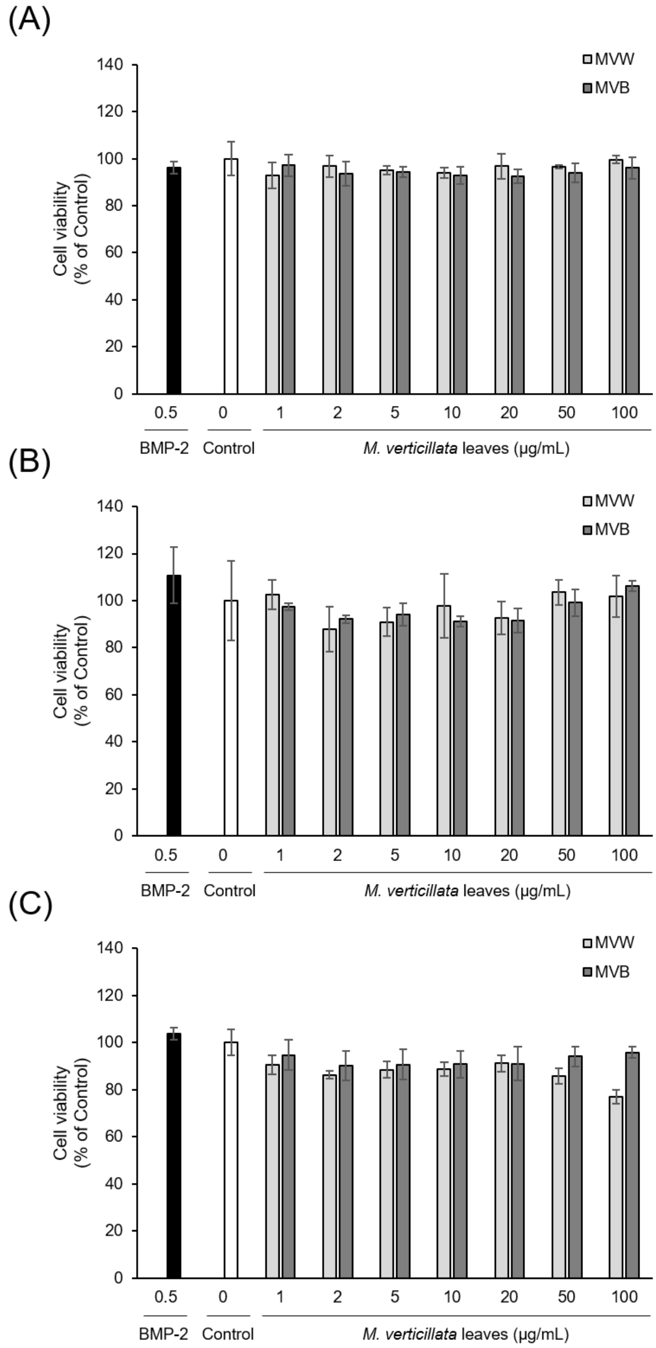

Supplementary Figure 3. Effect of MVW and MVB on the viability of C3H10T1/2 cells. The cells were seeded in 96-well plates, incubated for 24 h, then MVW or MVB was added to the medium and incubated for one to three days. Thereafter, MTT solution was added to the medium and incubated for 1 h. The medium was removed and DMSO was added to solve the MTT formazan. (A) 24 h, (B) 48 h, (C) 72 h.

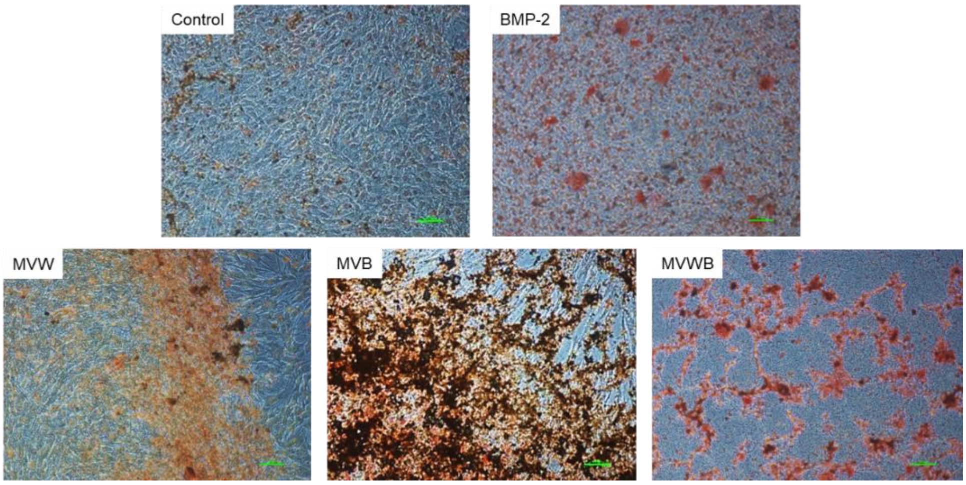

Supplementary Figure 4. Effect of MVW, MVB, and MVWB on the mineralization of C3H10T1/2 cells using Alizarin Red S staining. The intracellular mineralization was determined by staining the calcium deposits using Alizarin Red S dye. C3H10T1/2 cells were observed at 20 X magnification with a phase-contrast microscope, and red staining indicated the location and intensity of calcium deposits.

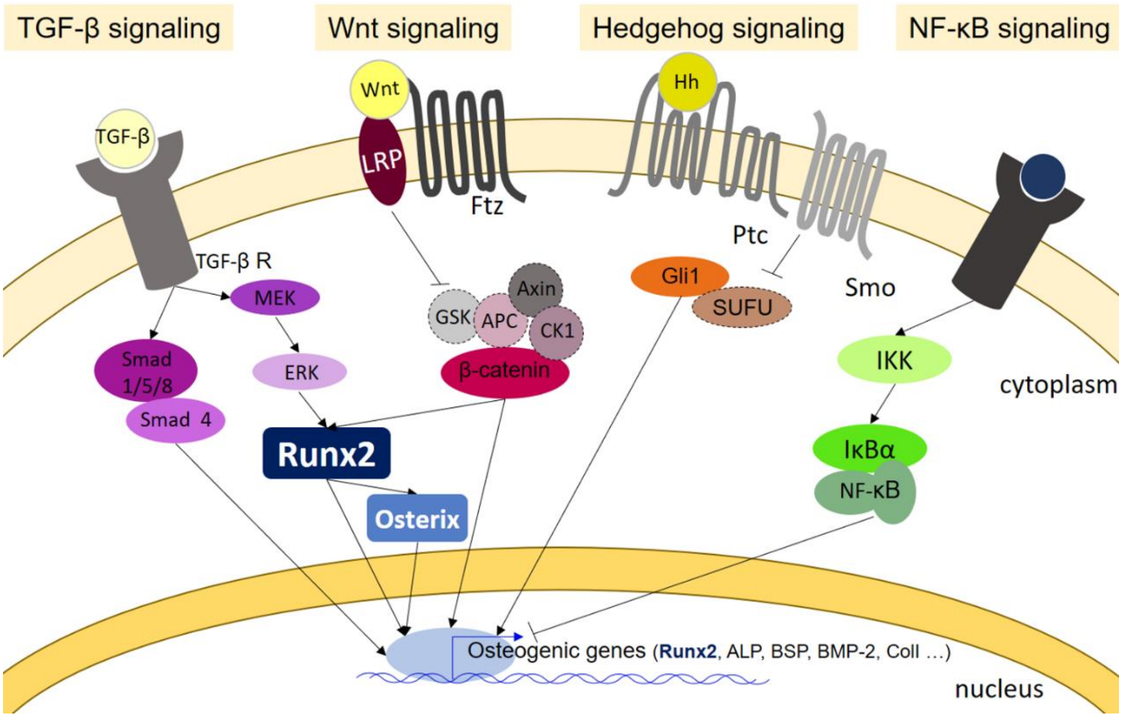

Supplementary Figure 5. Signaling pathways for osteoblast differentiation (modified from Huang et al. 2007).

Supplementary Table 1. Compounds detected by LC-MS analysis of the aqueous extracts of the *M. verticillata* leaves.

Peak No.	RT (min)	$[M-H]^-$ (*m/z*)	MS/MS fragments (m/z)	Area		Name of compound
				MVW	MVWB	
1	4.9	285.0383	285.0383, 165.0559, 118.9643	69,433	219,013	kaempferol
2	5.8	461.0724	461.0724, 285.0400	770,268	357,934	kaempferol-glucuronide
